# Evaluation of new suspension system for limb prosthetics

**DOI:** 10.1186/1475-925X-13-1

**Published:** 2014-01-10

**Authors:** Hossein Gholizadeh, Noor Azuan Abu Osman, Arezoo Eshraghi, Sadeeq Ali, Nooranida Arifin, Wan Abu Bakar Wan Abas

**Affiliations:** 1 Department of Biomedical Engineering, Faculty of Engineering, University of Malaya, Kuala Lumpur, Malaysia

**Keywords:** Transtibial prostheses, Prosthetic liner, Prosthetic suspension, Lower limb prosthesis, Below-knee prosthesis, Prosthetic socket, Amputees

## Abstract

**Background:**

Good prosthetic suspension system secures the residual limb inside the prosthetic socket and enables easy donning and doffing. This study aimed to introduce, evaluate and compare a newly designed prosthetic suspension system (HOLO) with the current suspension systems (suction, pin/lock and magnetic systems).

**Methods:**

All the suspension systems were tested (tensile testing machine) in terms of the degree of the shear strength and the patient’s comfort. Nine transtibial amputees participated in this study. The patients were asked to use four different suspension systems. Afterwards, each participant completed a questionnaire for each system to evaluate their comfort. Furthermore, the systems were compared in terms of the cost.

**Results:**

The maximum tensile load that the new system could bear was 490 N (SD, 5.5) before the system failed. Pin/lock, magnetic and suction suspension systems could tolerate loads of580 N (SD, 8.5), 350.9 (SD, 7) and 310 N (SD, 8.4), respectively. Our subjects were satisfied with the new hook and loop system, particularly in terms of easy donning and doffing. Furthermore, the new system is considerably cheaper (35 times) than the current locking systems in the market.

**Conclusions:**

The new suspension system could successfully retain the prosthesis on the residual limb as a good alternative for lower limb amputees. In addition, the new system addresses some problems of the existing systems and is more cost effective than its counterparts.

## Background

About 1.6 million individuals with limb loss lived in the United States according to 2005 statistics (about 0.05% of the community). This number is predicted to be doubled to 3.6 million by the year 2050 [1].

Non-use or limited use of prosthetic devices is concern for rehabilitation of amputees. Provision of good prosthesis is also the key element in the rehabilitation of persons with amputation. The amputee’s functional needs and his/her satisfaction with the prosthesis should be taken into account when selecting a suspension system [1-7].

Suspension systems and sockets are the most critical components of the prosthesis that are in direct contact with the amputees' residual limb. Excessive translation, rotation, and vertical movements between the residual limb and socket should be prevented through the suspension system [4,6-8]. A number of prosthetic suspension systems are available for lower or upper limb amputees [2,4,9,10]. Suspension systems mostly have two parts; namely, soft liner and lock system, which link them to the rest of the prosthetic components [11-13]. Objective and subjective studies have revealed improved suspension with the use of silicone liners among different systems in the market [4,9], most probably due to firm union between the socket and the residual limb. However, based on the literature, no standard suspension system exists that satisfy the needs of all amputees.

There are different methods to fix the silicone liner to prosthetic devices (lock system). Internal pin/lock systems, suction by hypobaric seals (seal-in liners) or sleeve, lanyard, and recently magnetic system (MPSS) [14] have been introduced in addition to the previous exterior systems [4,8,15,16]. The magnetic suspension system (MPSS) is used with the silicone liners as one of the most common soft interfaces. Thus, it incorporates a cap that is matched both to the liner’s distal end and the main body of the coupling device, with the dimensions comparable to the liner proportions. Coupling to the liner is enabled through a central screw. The magnetic power is developed through the body of the coupling that also intensifies the magnetic field by flanges. A mechanical switch controls the magnetic power. To reduce the risk of fall due to any failure of the coupling, an acoustic alarm system can be added as an optional accessory [14].

Researchers have targeted various determinants of satisfactory prosthetic services. Easy donning/doffing, fit, and low pistoning are the main variables that indicate proper prosthetic suspension [8,17]. Although silicone liners with the pin/lock or suction systems offer superior suspension for lower limb amputees [8,18], problems also arise from some of them [6,18]. Milking is observed in the pin/lock systems as the tissue is stretched where the pin is screwed to the liner during ambulation [19,20]. Moreover, it is challenging to use the system for amputees with long stumps or contractures. These problems could be solved by suction or vacuum system (seal-in or sleeve). In the seal-in system there is a hypobaric sealing membrane around the silicon liner, which increases surface contact with the socket wall and creates the suction inside the socket. In this system, there is no need to use external sleeve or shuttle lock mechanism to fix the liner to the socket [18]. Furthermore, suction systems can create better fit inside the socket and decrease the amount of vertical movement compared to other systems. Nevertheless, donning and doffing is the main concern, especially for elderly amputees [6,15-17]. Additionally, individuals should have good manipulation skills to don and doff the seal-in liner.

The main factors that should be taken into account when designing prosthetic suspension (soft liner and lock system) are safety, comfort, function, easy donning/doffing, durability, cosmetic appearance, and cost. Thus, to overcome some of the disadvantages of current prosthetic suspension systems, a new system was designed by the authors called HOLO (using hook and loop fabric as a lock system) to be used with silicone liners as they are widely available and commonly used. As the costs of these materials (hook and loop or Velcro) have decreased, their use has multiplied [21]. The hook is often referred to as the male portion, while the loop is referred to as the female portion. The best way to evaluate the strength of a hook and loop fastener is to measure shear strength [21].

Therefore, this study aimed to introduce and evaluate a newly designed prosthetic suspension system in terms of the degree of shear strength, and the patient’s comfort. Furthermore, comparisons of shear strength, amputee satisfaction and cost were made among the new system, the suction (seal-in x5 liner), locking (dermo liner), and magnetic MPSS [14] systems.

We were interested to know if the HOLO suspension system is easy to don and doff, creates proper fit inside the socket, and is lighter compared to the other systems. It was also intended to if the amputees will be more satisfied with this new system.

## Methods

**Figure 1:**
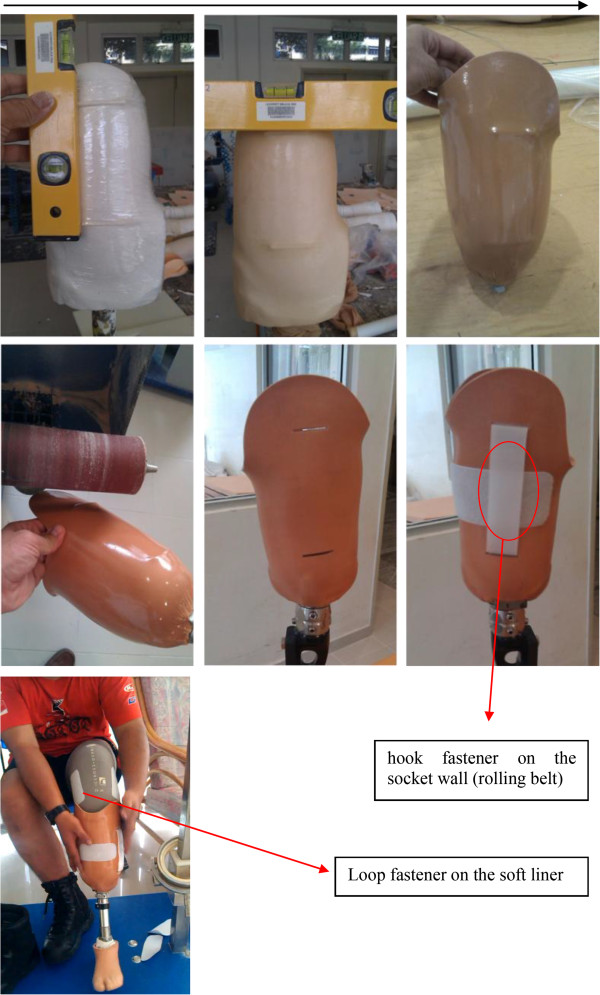
Fabrication process of new system (Hook and Loop system).

The hook and loop (Velcro) was used as main part of this suspension system to function as a lock. Two small openings were created on the socket wall (medial and lateral) in proximal and distal regions of the socket. The proximal opening was created below the knee center in the transtibial socket to avoid any limitation in knee flexion. The openings must be parallel and in the socket direction. Furthermore, we attached a small piece of hook (3 cm^2^) at the distal end of the socket. We used the hook fastener (Polyester Hook & Loop Velcro V-STRONG, 100% Polyester) on the socket wall (rolling belt) and the loop fastener on the soft liner (silicone liner) (Figure 1).

**Figure 2:**
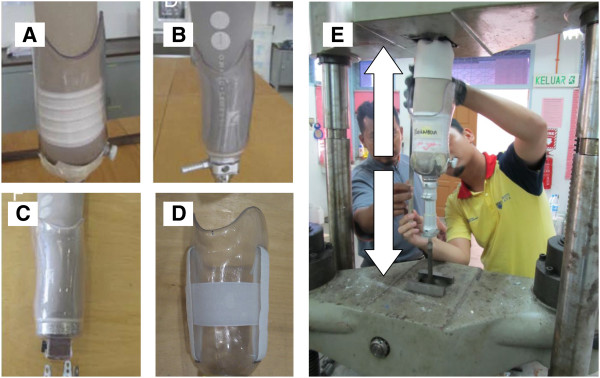
The prosthetic suspension systems used in this study: A (Seal-In X5), B (Pin/Lock system), C (Magnetic system), D (Hook and Loop); E (Mechanical testing under tensile loading).

The new suspension system was tested mechanically before testing on the subjects. Furthermore, we tested the other suspension systems used in this study to compare with the new design. Mechanical testing under tensile loading was performed using the universal testing machine INSTRON 4466 to find out how much tensile force each suspension system (lock mechanism) could tolerate before it fails (Figure 2). Four prosthetic sockets with different suspension systems were made by transparent thermoplastic material. We used the NorthPlex 12 mm (North Sea Plastics Ltd.) to check the movement between the silicone liner and prosthetic socket during the tensile loading test (Figure 2).

### Participants and experiment

**Figure 3:**
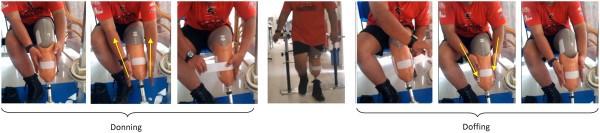
Donning and doffing procedures.

**Figure 4:**
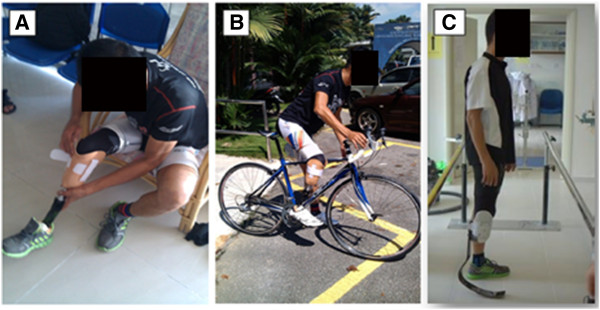
Subject 1 used the new system for walking (A), cycling (B) and running (C).

The study was approved by the Medical Ethics Committee, University of Malaya Medical Centre. Nine transtibial amputees participated in the study (Table 1). Following acquisition of written informed consent, each participant was provided with four transtibial prostheses (pin/lock, seal-in x5, magnetic (MPSS), and the new hook/loop (HOLO) suspension system) (Figure 2). To ensure consistent prosthetic quality, fabrication and aligning were done by a single prosthetist . All the subjects were fitted with a transparent check socket to ensure that the socket was Total Surface Bearing (TSB). Then, they were asked to walk with their new prostheses in the prosthetic laboratory (Department of Biomedical Engineering, University of Malaya, Malaysia) to become familiar with and adapt to the new sockets. All the subjects were given a trial period of at least 4 weeks (for each suspension systems) to become accustomed to the new prostheses (Figures 3 and 4).

**Table 1:** Characteristics of the participants

**Subject no.**	**Age**	**Height (cm)**	**Mass (kg)**	**Level of amputation**	**Cause of amputation**	**Time since amputation**	**Stump length (cm)**	**Mobility grade**	**Stump appearance and problem with own prosthesis**
1	39	170	65	TT	Trauma	5	14	K4	Bony and conical in shape. The bony end of the residual limb was painful during the swing phase of gait. He was using pin/lock system prior to the study.
2	23	167	82	TT	Trauma	3	15	K3	Cylindrical in shape. He was using PTB socket with Pelite (soft liner). He encountered numerous problems with prosthesis, such as pain, wound at the end of his stump and too much movement (pistoning) within the socket. Most of the weight was centralized at the end of the socket.
3	51	172	67	TT	Trauma	5	14	K3	Bony and conical in shape. The bony end of the residual limb and fibular head were painful during the swing phase of gait and while wearing the prosthesis. He was using pin/lock system prior to the study.
4	40	180	95	TT	Diabetes	7	16	K2	Cylindrical in shape. He was using pin/lock system prior to the study. He encountered difficulties in aligning the pin during wearing the prosthesis. He experienced a disorder in his left hand.
5	75	182	75	TT	Diabetes	8	13	K2	Bony and conical in shape. The bony end of the residual limb was painful during the swing phase of gait. He was using pin/lock system prior to the study.
6	45	185	84	TT	Trauma	26	12	K3	Short stump. He was using PTB socket with Pelite (soft liner). He had pain at the end of stump and too much movement (pistoning) within the socket. Most of his weight was centralized at the end of the socket.
7	41	173	95	TT	Trauma	5	14	K3	Cylindrical in shape. He was using pin/lock system prior to the study. He did not have any problem with his prosthesis.
8	34	175	78	TT	Trauma	10	28	K3	Cylindrical in shape. He did not feel any pain at the stump. He was using pin/lock system prior to the study.
9	32	163	72	TT	Trauma	18	25	K2	Conical in shape. Bony prominence was evident at the end of his stump. He did not feel any pain at the stump. He was using pin/lock system prior to the study.

Satisfaction with each suspension system was evaluated using a questionnaire and subjective feedback was also collected for each system. We used some parts of the prosthesis evaluation questionnaire (PEQ) to distinguish the perceptions of subjects towards the four suspension systems [22]. The questionnaire inquired about the ability to put on or take off the prosthesis, fit of prosthesis, ambulatory ability with the prosthesis on even and uneven grounds, ability to negotiate the stairs, satisfaction while sitting with prosthesis, complaints of the respondents about rotation and pistoning inside the socket, sweating, swelling, bad smell, irritating sound, pain and one question regarding the overall satisfaction with the systems. The rate of satisfaction was from 0 to 100 (“100” equal to “highly satisfactory”). Complaint scores of 0 indicated “highly bothering” and 100 meant “not bothering whatsoever”.

Qualitative analyses were performed on the respondents’ demographic data. We used SPSS 18.0 (IBM Corporation; Armonk, New York) for the data analyses, with the p-values set at 0.05. To evaluate amputees' satisfaction and problem with each system and to compare the systems, paired-samples t tests were used. Furthermore, we compared the cost of our new system with the common suspension system in the world (pin/lock systems).

## Results

### Mechanical test

The maximum tensile load that the new system could bear was 490 N (SD, 5.5). Moreover, movement within the socket was only 4 mm (between the liner and the socket) during 30 seconds of tensile loading. The pin/lock system could tolerate loading of 580 N (SD, 8.5); however, the lock system lost its function after three trials. The magnetic (MPSS) and seal-in (suction) could tolerate loads of 350.9 (SD, 7) and 310 N (SD, 8.4), respectively. With the pin/lock and magnetic system, there was no movement between the end of the liner and socket, and there were 18 and 12 mm of traction in the silicone liner, respectively. Furthermore, we observed 7mm of movement between the liner and socket with the seal-in liner before the system was failed.

### Subject characteristic

All the subjects in this study were males. Diabetes and trauma were the common causes of amputation. The mean age (year) and height (cm) of the participants were 42.2 (SD, 14.7) and 174.1 (SD, 7.2), respectively (Table 1). On average, the participants went through amputation 9.7 (SD, 7.5) years prior to the study. The average mass of prostheses (transtibial) for the magnetic (MPSS) suspension, pin/lock (Icelock 200 Series Clutch 4H 214), suction (seal-in x5), and the new HOLO system among the nine transtibial subjects were 1.89, 1.80, 1.65, and 1.60 kg, respectively.

### Questionnaire

Based on the PEQ results, the participants were generally pleased with the new system(Tables 2 and 3), and there was no significant difference with the pin/lock (*P* < 0.643) and magnetic systems (*P* < 0.672). However, there was a significant difference between the new system and suction system using the seal-in liner (*P* < 0.000). There was no significant difference between the HOLO and the other systems regarding the sitting, walking (even and uneven surface), climbing the stairs, sweating, swelling, and smell. The suction suspension system (seal-in liner) could create better fit compared to the other systems, and there was significant difference between the HOLO and suction systems (*P* < 0.002). Our subjects were happier with the new system (HOLO) due to easy donning and doffing procedures (*P* < 0.002). Also, there was significant difference with the pin/lock and suction systems (*P* < 0.000). The questionnaire revealed that the amputees experienced less pistoning or vertical movement, rotation and sound inside the socket with the suction socket (seal-in) compared to the other systems. However, the new system (HOLO) creates more sound compared to the other systems. The irritating sound with the new system was only created during doffing the prosthesis (tearing noise from the Hook and Loop) (Table 3). The respondents also mentioned that the prosthesis with the suction (seal-in) made them to feel the artificial limb as a natural body part (subjective feedback). Moreover, there was no traction or pain at the distal liner.

**Table 2:** Subjective feedback of the participants

**Subject no.**	**Subject’s preference**		**Mobility grade**	**Subjective feedback**
1	Seal-In	1	K4	He did not feel any pain at the distal of his residual limb with the Seal-In and the new suspension system during walking. He experienced more confidence and also stated that the Seal-In was more suitable than the other suspension systems. While removing the prosthesis was more challenging, he preferred to use the seal-in system.
	Pin/Lock	4		
	Magnetic	2		
	Hook/Loop	3		
2	Seal-In	4	K3	He was more satisfied with the silicone liners compared to the PTB with Pelite liner. After changing to silicone liner (TSB socket), he did not have pain at the distal end of the residual limb, and the wound was healed after two weeks. He felt more confident with the silicone liner and different lock systems (pin/lock, magnet or HOLO). Among the four systems in this study, he preferred the HOLO, the magnetic system, and the pin/lock system.
	Pin/Lock	3		
	Magnetic	2		
	Hook/Loop	1		
3	Seal-In	4	K3	He did not feel any pain at the distal of residual limb with the seal-in and the new suspension system. However, he had pain during donning and doffing with the seal-In liner. He stated that the seal-in was more suitable during walking, but wearing and removing the prosthesis was extremely more difficult compared to the other suspension systems.
	Pin/Lock	3		
	Magnetic	1		
	Hook/Loop	2		
4	Seal-In	4	K2	It was very difficult to use the seal-In due to upper limb weakness; he preferred the hook and loop, pin/lock and magnetic systems mostly due to easy donning and doffing.
	Pin/Lock	2		
	Magnetic	3		
	Hook/Loop	1		
5	Seal-In	4	K2	He did not feel pain with the seal-in and the new suspension system. Nevertheless, he preferred the new suspension system due to its advantages of easy donning and doffing. He was not happy with the tearing noise during doffing the prosthesis.
	Pin/Lock	3		
	Magnetic	2		
	Hook/Loop	1		
6	Seal-In	4	K3	Pain at the end of the socket was less with the TSB socket compared to the PTB socket. He was satisfied with the pin/lock, hook/loop and magnetic systems while he felt more socket fit and less rotation inside the socket with the seal-in. He mentioned that he is not going to use the seal-in due to the difficulty in donning and doffing.
	Pin/Lock	1		
	Magnetic	3		
	Hook/Loop	2		
7	Seal-In	3	K3	He felt more socket fit and higher confidence with the seal-in during walking but he was not satisfied with the donning and doffing procedures. He preferred to use the pin/lock and magnetic systems. He was not happy with the hook/lop system due to the sound developed during doffing the prosthesis.
	Pin/Lock	1		
	Magnetic	2		
	Hook/Loop	4		
8	Seal-In	4	K3	He was happier with the pin/lock and HOLO systems due to easy donning and doffing procedures. Also, he felt less traction at the end of the socket with HOLO and seal-in system.
	Pin/Lock	1		
	Magnetic	3		
	Hook/Loop	2		
9	Seal-In	4	K2	He felt more comfortable at the distal end with the seal-in and the new suspension system, and he was more confident during walking. Regarding the donning and doffing, he preferred the pin/lock and HOLO system. He chose the pin/lock as his first choice due to easy donning and doffing.
	Pin/Lock	1		
	Magnetic	3		
	Hook/Loop	2		

**Table 3:** Satisfaction and problems with different suspension systems

**Descriptive statistics**			**Paired-samples t test**		
**Questionnaire item**	**Suspension system**	**Mean**	**Suspension system**		**Sig. (2-tailed)**
Fitting	Pin/lock	75.59	Holo	Pin/lock	.125
	Seal-In (suction)	87.09		Seal-In (suction)	.002*
	Magnetic	76.84		Magnetic	.181
	Holo	80.50			
Donning/ Doffing	Pin/lock	71.10	Holo	Pin/lock	.002*
	Seal-In (suction)	57.89		Seal-In (suction)	.000*
	Magnetic	79.65		Magnetic	.080
	Holo	83.30			
Sitting	Pin/lock	79.40	Holo	Pin/lock	.767
	Seal-In (suction)	79.45		Seal-In (suction)	.666
	Magnetic	78.40		Magnetic	.360
	Holo	80.20			
Walking	Pin/lock	77.10	Holo	Pin/lock	.089
	Seal-In (suction)	78.30		Seal-In (suction)	.906
	Magnetic	79.03		Magnetic	.452
	Holo	78.50			
Walking (uneven surface)	Pin/lock	75.00	Holo	Pin/lock	.415
	Seal-In (suction)	76.80		Seal-In (suction)	.771
	Magnetic	77.80		Magnetic	.152
	Holo	76.10			
Walking (stairs)	Pin/lock	75.40	Holo	Pin/lock	.102
	Seal-In (suction)	77.63		Seal-In (suction)	.773
	Magnetic	78.20		Magnetic	.588
	Holo	77.30			
Sweating	Pin/lock	67.83	Holo	Pin/lock	.106
	Seal-In (suction)	65.30		Seal-In (suction)	.308
	Magnetic	63.40		Magnetic	.950
	Holo	63.30			
Pistoning	Pin/lock	84.13	Holo	Pin/lock	.020*
	Seal-In (suction)	96.40		Seal-In (suction)	.000*
	Magnetic	78.80		Magnetic	.681
	Holo	79.30			
Rotation	Pin/lock	80.19	Holo	Pin/lock	.002*
	Seal-In (suction)	99.50		Seal-In (suction)	.000*
	Magnetic	82.09		Magnetic	.172
	Holo	83.50			
Swelling	Pin/lock	85.89	Holo	Pin/lock	.800
	Seal-In (suction)	88.50		Seal-In (suction)	.080
	Magnetic	83.63		Magnetic	.173
	Holo	85.50			
Smell	Pin/lock	84.70	Holo	Pin/lock	.569
	Seal-In (suction)	83.10		Seal-In (suction)	.298
	Magnetic	84.60		Magnetic	.745
	Holo	85.40			
Sound	Pin/lock	70.30	Holo	Pin/lock	.218
	Seal-In (suction)	96.50		Seal-In (suction)	.000*
	Magnetic	80.15		Magnetic	.000*
	Holo	66.90			
Pain	Pin/lock	76.30	Holo	Pin/lock	.000*
	Seal-In (suction)	80.65		Seal-In (suction)	.003*
	Magnetic	86.70		Magnetic	.723
	Holo	87.40			
Overall satisfaction	Pin/lock	83.40	Holo	Pin/lock	.643
	Seal-In (suction)	64.60		Seal-In (suction)	.000*
	Magnetic	79.60		Magnetic	.672
	Holo	80.20			

*Significant differences.

The cost of pin/lock system ranged from 50 to 300 USD (http://www.oandp.org/publications/resident/pdf/Locks.pdf) [23], while our new system costs less than 5 USD (35 times cheaper than the average price of pin lock systems).

## Discussion

Prosthetists need to decide whether a suspension system is suitable or not for residual limb length, shape (i.e., cylindrical or conical), muscle strength, soft tissue, bony prominence, pain, aspiration of amputee, level of activity, upper limb strength and amputee’s financial situation. In this study, we introduced and tested a new simple method for suspending the residual limb within the prosthetic socket. Furthermore, we compared our new system with three different prosthetic suspension systems to examine the maximum tensile load that each system could bear, and their effects on patient’s satisfaction.

### Mechanical test

Based on the literature, load of 30 N to 50 N was applied to the prosthetic leg (suspension system) in the swing phase of gait. In each gait cycle, this amount of load was applied to the suspension system in less than one second during the swing phase [24]. One of the factors that increase this load is weight of prosthesis that can influence the amputee’s satisfaction with the device [17], especially in children and elderly amputees. The results show that the prosthesis could be made lighter by using the HOLO system. The magnetic (MPSS), pin/lock, and seal-in suspension systems were heavier than our new system by 15.3%, 11.1%, and 3%, respectively.

Among the four systems tested in this study, the pin/lock system could tolerate the highest loading (580 N). Our new suspension system could bear 490 N of tensile loading before it failed, which is almost 10 times more than the applied load in normal walking. This test proved that the safety of our system is similar to that of other suspension systems. Even after applying large amount of load, only 4 mm of vertical movement occurred within the socket with the new system (between the liner and socket walls) during the 30 seconds of tensile loading. The lesser movement in this new system is comparable to the magnetic (MPSS) [14] and the pin/lock systems, and can be attributed to the full attachment between the liner (loop) and socket walls (hook). Low movement inside the prosthetic socket has significant effect on the prosthesis function and amputee’s satisfaction [6,8,15].

### Questionnaire

According to the literature, researchers mostly use the PEQ to evaluate different prosthetic components. This questionnaire has good reliability and validity [10,22]. In this study, we only used some parts of this questionnaire. In the pin/lock systems, the soft liner (silicone) is connected to the prosthetic socket only by a distal pin; thus, the amputees feel traction and pain at the end of the stump, mainly during the swing phase of gait [8,16,18,20]. Moreover, they feel a lesser degree of fit inside the socket compared to the seal-in liner (suction). Nevertheless, the amputees are mostly satisfied with this system due to easy wear and removal of the prosthesis (Table 3).

The ease of donning and doffing may affect the prosthesis use tremendously, especially in the case of night-time toilet habits [6,15,18,25]. Gholizadeh et al. mentioned that firm attachment between the soft liner and socket walls in the seal-in liners could create a good feeling of confidence for amputees during walking [6,18]. Nevertheless, donning and doffing can be a challenging task, particularly for older amputees or for those with upper limb problems, such as stroke patients [2,8,15,26]. In the HOLO, the soft liner is attached to the socket walls similar to the Seal-In liners (firm attachment); nevertheless, donning and doffing is as easy as the pin/lock or magnetic systems [6,15]. Moreover, due to proper fitting inside the socket as well as less rotation and pistoning, our subjects were more satisfied during ambulation with the seal-in and HOLO suspension systems. Even if the volume of the stump is decreased, the amputees are able to use elastic socks (with loop fabric) over the silicone liner.

Furthermore, using the pin/lock or magnetic (MPSS) system for amputees with long stumps (transtibial, transfemoral or knee disarticulation) can be difficult. Also, if amputee has contracture in stump, aligning the pin is challenging. With this new suspension method (HOLO), which is similar to the suction (using the seal-in or sleeve), it is not necessary to provide extra space at the end of the socket. Moreover, amputees can wear the prosthesis even if they have contracture in residual limb.

This lock system (hook and loop) is much cheaper compared to the current available systems, and is accessible easily everywhere [23]. This new system can be a good choice for developing countries or children who need to change their prosthetic legs more than once a year. The only problem with the use of Velcro was the tearing noise during the removal of the liner from the socket (unfastening). This issue could be addressed by the use of less noisy types of Velcro.

Although the system could solve some prosthetic problems, it should be acknowledged that these findings may apply to other amputees only if evaluated on a larger sample size. Furthermore, the results of this study are considered as short-term effects of different prosthetic suspension systems; therefore, we believe that effect of this new system should be monitored in long term.

## Conclusions

Our study revealed that the new suspension system is a good alternative for individuals with transtibial or transfemoral amputation as it could solve some problems with the current systems. This system may have some advantages for amputees including ease of donning/doffing, firm attachment to the socket, low weight and low cost. Further research is needed to evaluate more amputees (upper and lower limbs), and to prepare a guideline for selection of suspension system.

## Abbreviations

Cm: Centimeter; HOLO: Hook and loop; Kg: Kilogram; Mm: Millimeter; MPSS: Magnetic prosthetic suspension system; N: Newton; PEQ: Prosthesis evaluation questionnaire; SD: Standard deviation.

## Competing interest

The authors have no conflict of interest.

## Authors’ contributions

HG designed the system and the protocol, fabricated the prostheses, conducted the experiments, collected and analyzed the data, discussed the results and drafted the manuscript. NAAO and WABAO supervised the overall project, and helped in revising the manuscript. AE and SA collected and analyzed the data, discussed the results, prepared some parts of the manuscript. NA conducted mechanical testing, analyzed the data and discussed the results. All the authors reviewed the manuscript. All authors read and approved the final manuscript.
